# The integrated health service model: the approach to restrain the vicious cycle to chronic diseases

**DOI:** 10.1186/s12913-019-4179-x

**Published:** 2019-05-31

**Authors:** Netsanet Fetene Wendimagegn, Marthie Bezuidenhout

**Affiliations:** 1 Yale Global Health Leadership Institute(GHLI), P.O. Box: 5874, Addis Ababa, Ethiopia; 2 University of South Africa, UNISA, Pretoria, South Africa

**Keywords:** Integrated health service, Chronic non-communicable diseases, Health promotion, Disease prevention

## Abstract

**Background:**

In life time, nearly each person succumbs to some sort of chronic disease and many develop complicated chronic diseases. It is critical to focus on preventive services with a relatively high health impact and favorable cost effectiveness. During routine health facility visits, it is advisable to evaluate both symptomatic and asymptomatic patients for their needs of health promotion and disease prevention services. This necessitates the development of an integrated health service (IHS) approach that incorporates health promotion, disease prevention and curative services.

**Methods:**

There were two phases for the study. The first phase explored the degree of promotive and preventive health care delivery at the health centers and hospitals. Phase two, utilizing the Delphi strategy, centered on looking for agreement on the finding from phase 1 and on IHS approach. Delphi questions were created based on the results of phase 1, and the reply choices were tied to a five point Likert scale. Consensus was considered come to when 75% of the experts concurred on an issue. From that point, advance clarification and agreement was looked for by implies of a second-round assessment for scores between 50 and 75%. Agreement on proposed IHS model, application of case finding and Periodic Health Examination (PHE) approaches were also sought. This study focuses on finding from phase 2.

**Result:**

Of the twenty experts, 90% (*n* = 18) agreed that the IHS framework shows the causal relationship of diseases and included plausible intervention approaches. Experts reached consensus (90%;*n* = 18) that case finding testing,screening patients for conditions other than the medical care they sought at a particular time, can be performed at health facilities. All experts (100%; *n* = 20) recommended conducting periodic health examinations in selected diseases for patients who are apparently not sick.

**Conclusion:**

The Integrated Health Service (IHS) framework was agreed by experts to be a plausible method in describing the causal relationship of chronic non-communicable, communicable, and nutrition-related diseases. The framework can play a vital role by preventing the acquiring, progression, suffering or dying from diseases through restraining the vicious cycle of chronic diseases.

**Electronic supplementary material:**

The online version of this article (10.1186/s12913-019-4179-x) contains supplementary material, which is available to authorized users.

## Background

The relatively small number of risk factors that account for a large share of the disease burden shows the need to take advantage of designing strategies that decrease or eliminate the major risks to health [1]. In life time, nearly each person succumbs to some sort of chronic disease and many develop complicated chronic diseases. For this reason, a convincing approach that recognizes the reality and impact of chronic disease,works to prevent and control it over the lifetime and maintain healthy life style is essential [2]. It is important to select interventions that are cost effective and can produce the maximum possible health improvements with limited resources. Therefore, both symptomatic and asymptomatic patients seeking health care should be assessed for the need of health promotion and preventive services during routine clinic health care visits [3].

All clinic contacts, whether for acute, chronic or preventive reasons, are valuable opportunities for health promotion and disease prevention activities to take place [3]. Moreover, it would be a loss of opportunity for reducing preventable diseases burden and for decreasing the cost of health care if preventative services are not provided to patients [4].This is particularly important for most chronic diseases.

Screening is the identification of unrecognized diseases or risk factors by means of history taking (e.g., asking if the patient smokes), physical examination (e.g., a blood pressure measurement), laboratory test (e.g. checking for proteinuria in a diabetic), or any other relevant procedure (e.g., a bone mineral density examination) that can be applied reasonably rapidly to asymptomatic people. Screening tests differentiate between apparently well persons (for the condition of interest) who have an increased likelihood of acquiring a disease, or a risk factor for a disease from people who have a low likelihood. Screening tests are part of all secondary, some primary and tertiary preventive activities [5]. Recommendations on disease types and timing for screening vary from country to country, based on the context and evidence-based findings. For instance, while the optimal interval for screening adults for hypertension is not clearing defined according to the USPSTF guideline [6], the Joint National Committee on the Prevention, Detection, Evaluation, and Treatment of High Blood Pressure recommends screening every 2 years with BP < 120/80 and screening every year with Systolic Blood Pressure (SBP) of 120–139 mmHg or Diastolic Blood Pressure (DBP) of 80–90 mmHg [7].

The case finding approach during patients’ routine hospital and clinic visits is the paradigm of viewing patients’ health needs beyond their presenting illness. Health service providers must assess the patient and apply the necessary preventive services required by each patient as identified during routine medical care [3]. Preventive medicine requires the health service provider to be proactive, the instigator of questions, screening, and treatment [8].

The periodic health examinations (PHEs) strategy is another health promotion and disease prevention approach. Patients who use periodic health examinations more likely obtain the advocated preventive health care services than patients who receive services during routine clinical care visits [4]. Periodic health examinations make contributions to the improvement of physician–patient relationship and give time for counselling, addressing patient expectations, and enhancing early detection of diseases. Patients hope to undergo counseling on health habits and risk factors for NCDs during a PHE along with a physical examination and various health screening tests. A comprehensive PHE may also be an opportunity for identification of several essential diagnoses or risk factors that cannot be recognized through routine screening. The PHE that consists of a comprehensive medical history, physical examination and testing is a valuable practice in outpatient department [9].

Both case finding and periodic health examination approach are critical in providing integrated health care services of chronic diseases. The four common risk factors in chronic diseases are tobacco use, harmful use of alcohol, an unhealthy diet, and lack of physical activity. If these risk factors are identified early in affected patients and properly dealt with, then the mortality and morbidity from chronic diseases can be reduced [10]. It is important for national action to address the social determinants of health and prevent the risk factors to the four common non-communicable diseases; cancer, cardiovascular diseases, chronic obstructive diseases and diabetes. The three highly prioritized interventions for these diseases, tobacco control, salt reduction, and the management of people at high risk of heart attack or stroke are anticipated to 25% reduce the premature NCDs mortality rate by 2025 [11].

Given the fact that chronic diseases causes illness for an extended amount of period and interrelated with infectious diseases, a comprehensive and coordinated health care approach is required. Today, scientists and physicians widely acknowledge the infective agent causes for some chronic diseases, like hepatitis virus related to chronic liver diseases and hepatocellular cancer [12]. The co-occurrence of infectious and chronic NCDs (double burden of diseases), necessitates integrated diseases prevention and management that ought to originate within the PHC sector professionals [13]. Matheson et al. [2] emphasize the short of community-based diseases prevention centers that can be directly accessed by anyone seeking to take care of or improve their health. Despite some existing rehabilitation facilities and life-style units in PHC centers, there are not any programs on a population-wide scale that target behavioral modification that include physical activity, exercise or alternative life-style choices. Nonetheless, each promising changes in risk factors and also the introduction of treatment contribute greatly to reducing mortality rates from chronic diseases. Moreover, evidences from policy modeling efforts strongly advises that any mortality reduction from chronic diseases may be realized if more aggressive targets for eliminating risk factors within the population are met and evidence-based treatment are applied [14]. Such call necessitates the development of an integrated health service (IHS) approach that incorporates health promotion, disease prevention and curative services.

Considering the country specific context, an integrated approach seeks to deal with health problems by means of providing case by case health care services in a very complete and integrated manner [15, 16]. Consequently, there is a developing preference for an integrated approach in preventing NCDs, infectious and nutrition connected illnesses that may suitably be applied in low- and middle-income countries (LMICs) [17].The Integrated Health Service Framework (IHS framework), modified from the linear “causation framework” [18] by the investigator, is regarded as a comprehensive and desirable theoretical framework for promoting health and preventing diseases. The “causation framework” successfully explains the determinants of health and their effect on NCDs [18–20].

**Fig. 1:**
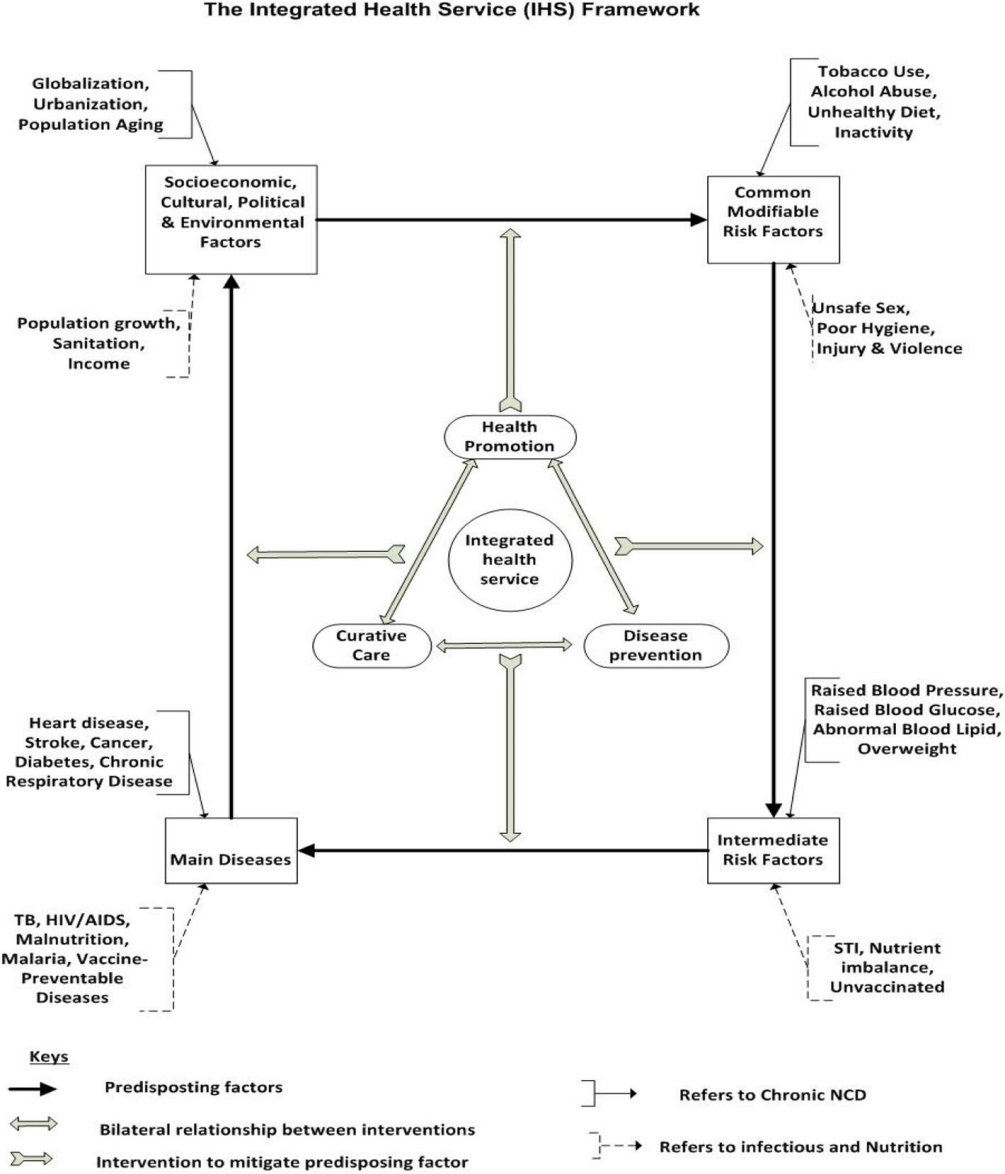
The Integrated Health Service (IHS) Framework (Adapted from: Bonita et al., 2006:103)

The “causation framework” describes the linear relationship of NCDs with intermediate risk factors, modifiable risk factors and the socio-economic, cultural and environmental contributory factors. Nevertheless, this model falls short of revealing the continuous relationship of predisposing factors and diseases as well as the intervention approaches necessary to avoid the risk factors and treat the diseases. In addition, the model does not include diseases other than chronic NCDs such as infectious and nutrition related diseases. Thus, there is a need for an Integrated Health Service (IHS) framework (modified by the researcher) which can address the relationships among predisposing factors along with the type of interventions needed in every step. The IHS framework applies to both communicable and non-communicable diseases as well as acute and chronic diseases (see Fig. 1).

The IHS included the Common modifiable risk factors to chronic diseases, like tobacco use, alcohol abuse, unhealthy diet and physical inactivity, that predispose to intermediate risk factors such as raised blood pressure, raised blood glucose, abnormal lipid level and overweight [18]. The attributable global deaths from the leading NCD risk factors are: 13% from raised blood pressure, 9% from tobacco use, 6% from raised blood glucose, 6% from physical inactivity, and 5% from overweight and obesity [21].The IHS framework considered the four particular behaviours, tobacco use, physical inactivity, unhealthy diet and the harmful use of alcohol that lead to four key metabolic (physiological) changes: raised blood pressure, overweight/obesity, hyperglycaemia and hyperlipidaemia, which are the intermediate risk factors for chronic diseases [21]. The intermediate risk factors are the cause of common chronic diseases such hypertension, diabetes, heart and respiratory diseases [18].

The IHS includes the leading causes of disease burden in low- and middle-income countries that comprise risk factors prevalent among the poor and are associated with malnutrition, unsafe water, sanitation, and hygiene, indoor smoke from household use of solid fuels, and unsafe sex [1]. The framework includes the human behaviour, often dictated by social and economic reasons that can influence the risk of infectious diseases (such as malaria, HIV and TB) for individuals and communities. For malaria, the major reasons for increased risk for acquiring malaria include cost of prevention, inconvenience to use medicines or insect repellents, or a lack of knowledge [22]. The factors that increases risk for HIV infection such unprotected sex with multiple partners and persons being treated for sexually transmitted are considered in the framework [23]. The two risk factors for malnutrition are classified as disease-related factors (that reduce intake despite the availability of foods) and inadequate availability of food (quality or presentation of food) which reduces intake are included in the framework [24].

Health and wellness are influenced by socio-economic, cultural, political and environmental factors. A healthy environment gives people the opportunity to make healthy choices and decreases their risk for heart disease, cancer, obesity, diabetes, respiratory diseases such as asthma, and injuries [25]. The IHS framework clearly demonstrates the relationship between the socio-economic, cultural, political and environmental factors, such as globalisation and population growth with the modifiable risk factors and how diseases lead to poor economic, social and environmental conditions which completes the vicious cycle. The high prevalence of diseases such as NCDs, HIV, malaria, malnutrition and vaccine preventable diseases in turn predisposes to weak socio economic conditions.

The IHS framework also demonstrates how health promotion, disease prevention and treatment interventions should be integrated to mitigate the continuous relationships of predisposing factors. Increasing interventions against joint risk factors for NCDs and against a major infectious disease through fighting malnutrition, tobacco and alcohol use is a priority. Preventing common cancers by immunizing against the virus that brings them is another priority [13]. Early detection of people at high risk of NCDs can be conducted through community outreach diagnostic testing approach. Likewise, treatment and prevention services can be carried out in the community through nurses and health extension workers with minimal dependence on clinical staff, particularly clinicians; which can be rapidly scaled up [26].

The IHS framework postulates that the vicious cycle keeps on unless the health promotion and disease prevention services are integrated into the curative domain to stop its continuation. The aim of the study was to explore the appropriateness of using an Integrated Health Service (IHS) framework in hospitals and health centres for provision of integrated promotive, preventive and curative health care services to patients seeking medical assistance.

## Methods

There were two phases for the study. The first phase explored the degree of promotive and preventive health care delivery at 22 selected public health centers and hospitals found in Addis Ababa. The study applied a quantitative research paradigm in which both an exploratory and descriptive research design were utilized. A multi-stage sampling technique was applied to cluster the government health facilities into public general hospitals and health centers; then, simple random sampling technique was used to further select health facilities. Systematic sampling was applied to select patients for interview after receiving health care services at outpatient and inpatient medical department of selected health facilities. Health facility managers were administered with questionnaire that was designed to capture the health facility’s readiness for addressing the common modifiable risk factors and intermediate risk factors for chronic diseases as well as treating patients with common chronic diseases. Data-collection instruments were pre-tested after which data was collected from 848 patients leaving outpatient and inpatient medical departments and from 22 health facility managers by means of a structured interview schedule.

Phase 2, using the Delphi consensus seeking process, focused on validation of the finding from phase 1 and developing an integrated health service framework for the improvement of health promotion and disease prevention services at hospitals and health centres. The Delphi technique is well suited as a method for consensus-building by using a series of questionnaires and multiple iterations to collect data from a panel of selected subjects [27].The Delphi technique was used as a means of reaching consensus among experts in the field of clinical and public health, in terms of provisional recommendations based on the findings of phase 1.

Phase 2 applied the Delphi consensus seeking method, focused on validation of the phase 1 finding and reaching agreement on an integrated health service framework developed for improvement of health promotion and diseases prevention services at hospitals and health centres. The Delphi technique is an appropriate method for consensus-building on subjects involving experts applying series of questions and multiple reiterations [27]. Twenty experts in areas of clinical and public health, worked in health promotion and diseases prevention field for more than 10 years in Ethiopia, participated in the two rounds of Delphi discussions (Table 1). Delphi questions were created based on the results of phase 1, and the reply choices were tied to a five point Likert scale; weighing if respondents reach agreement on each questions (Additional file 1). Consensus was considered come to when 75% of the experts concurred on an issue [28]. From that point, advance clarification and agreement was looked for by implies of a second-round assessment for scores greater than 50% but less than 75% (Additional file 2). This gave opportunity for experts who scored in this range to review other experts agreement and explanations to reconsider their own scores. In Delphi technique examining the responses from the panel members and including their feedback in subsequent rounds also motivates them for more active participation [28]. Both round questions demanded experts’ opinions and propositions on the issue presented. Finally, the response options of “agree” and “strongly agree” were categorized as positive “agree” reply. This study focuses on phase 2 findings but included some findings necessary to draw recommendations from phase 1 [16].

## Result

The first section of result includes the overall experts consensus on recommendations from phase 1 and phase 2 findings while the second part focuses experts agreement on IHS framework.

Screening is a process of detecting apparently healthy people who may be at increased risk of a disease or health state [29]. In this study, screening service provision was classified as a case finding approach that is integrated in the routine health care service and as a periodic health examination that is conducted every 1–2 years. The main reason for integration of screening for certain diseases in the routine health care service was to use patients’ presence as an opportunity for disease detection. It should be noted, however, that the type of screening services suggested by the experts to be provided as a case finding service can also be provided during periodic health examinations. For example, screening for hypertension and its complications; cervical and breast cancer, and STI screening and counselling.

### Case finding

Case finding refers to testing or screening of patients for conditions other than ones for which they sought medical care. According to Dermot et al. [30], case finding among people attending primary health care services is a central part of PHC’s contribution to NCD control. The importance of finding cases during routine health care for a particular gender, age group and disease, such as hypertension, diabetes, cervical cancer, tuberculosis, obesity, breast cancer and STI, can lead to early diagnosis and preventative measures or treatment before disease progression can take place. Case finding can be provided as part of routine health care or during physical examination. The experts reached consensus (90%,*n* = 18) that case finding testing or screening of patients for conditions other than the medical care they sought at a particular time can be performed using the opportunity of patients’ presence at health facilities. The experts agreed on the plausibility of case finding in view of hypertension, diabetes, cervical cancer, breast cancer, HIV and TB, and for patients at high risk for STI (see Table 2).

**Table 1:** Delphi experts’ agreement on performing case finding testing/screening for certain diseases (*n* = 20)

Experts agreement on case finding and the screening activities (first round)	n	Percent
Experts’ agreement on applying case finding approach as a routine health care activities	18	90.0
Hypertension and related complications	18	90.0
Cervical cancer	18	90.0
Diabetes	17	85.0
HIV	17	85.0
TB	16	80.0
Obesity	12	60.0
Smokers	12	60.0
Physical inactivity	11	55.0
COPD (chronic Obstructive Pulmonary Disease)	10	50.0
Harmful alcohol users	9	45.0
Screening activities in case finding (second round)	n	Percent
Actively searching for obesity	8	40.0
Finding smokers to provide counselling and care	7	35.0
Actively looking for physical inactivity	7	35.0
Actively looking for patients at risk of breast cancer	16	80.0
Actively finding patients at high risk for STI	15	75.0

**Table 2:** Delphi round Participants Profile

S.N	Age range	Profession	Education	Qualification area	Experience range (Year)
1.	40–44	Public Health specialist	Nurse, MPH	NCD, Hospital patient care, PHC, HIV, Nutrition, EPI	15–19
2.	35–39	Research Advisor	Health officer, MPH, PHD	NCD, EPI, HIV, RH, Research, M&E	10–14
3.	45–49	General Practitioner	Medical Doctor	NCD, hospital patient care, EPI, HIV, Maternal health	20–24
4.	35–39	Public Health specialist	Medical officer, MPH	NCD, EPI,RH,PHC	10–14
5.	35–39	Ophthalmologist	MD, Ophthalmologist	NCD, hospital patient care, Ophthalmology	10–14
6.	40–44	Senior Nurse	Nurse, MSc	NCD, Nursing care, EPI,HIV,RH	20–24
7.	35–39	Adviser	MD,MPH	NCD	10–14
8.	35–39	Public Health specialist	Health officer, MPH	NCD, PHC,HIV, Nutrition	10–14
9.	45–49	Nurse	BSC	NCD, PHC,HIV, Nutrition, RH, Nursing care	30–34
10.	35–39	Nurse	BSC	NCD, PHC,HIV, Nutrition, RH, Nursing care	10–14
11.	33–39	Nurse	BSC	NCD, PHC,HIV, Nutrition, RH, Nursing care	10–14
12.	35–39	Public Health specialist, MPH	BSC, MPH	NCD, EPI,HIV, RH, Research, M&E	10–14
13.	50–54	General Practitioner	MD,MPH, Associate professor	NCD, Hospital patient care, EPI, HIV, RH, Nutrition, Research, M&E	15–19
14.	35–39	General Practitioner	Medical Doctor	NCD, Hospital patient care, HIV	10–14
15.	45–49	Public Health specialist, MPH	BSC, MPH	NCD, PHC,HIV, Nutrition	15–19
16.	35–39	General Practitioner	Medical Doctor	NCD, Hospital patient care, laboratory	10–14
17.	60–64	Public Health specialist	BSC, MPH	NCD, Nursing care, EPI,HIV,RH	35–40
18.	45–49	Nurse	BSC	NCD, PHC,HIV, Nutrition, RH, Nursing care	20–24
19.	35–39	Public Health specialist, MPH	Health officer, MPH	NCD, PHC, Nutrition	10–14
20.	35–39	Public Health specialist, MD	Medical Doctor, MPH	NCD, hospital patient care, PHC,RH,EPI	15–19

**Table 3:** Delphi first round, experts’ agreement on specific screening activities during periodic health check-up areas (*n* = 20)

Experts agreement on periodic check-ups and the screening activities (first round)	n	Percent
Experts’ agreement on having periodic health check-ups in Ethiopia	20	100.0
Including periodic health check-ups in community or individual insurance system	19	95.0
Screening for hypertension and its complications	19	95.0
Screening for diabetes for patients with hypertension or BMI > 25	19	95.0
Measuring blood cholesterol level and counselling on healthy diet and obesity	17	85.0
Pap smear screening every 3 years beginning age 21	17	85.0
Clinical breast examination every 1–2 years for women over 50 years	16	80.0
STI screening and counselling	16	80.0
Visual acuity screening using Snellen sight chart	16	80.0
Counselling on physical activity	15	75.0
Vaccination for HPV for both sexes between age 9–26 years	13	65.0
Screening on road safety and counselling on seat-belt use, drinking and driving	13	65.0
Screening mammography examination every 1–2 years for women over 40 years	12	60.0
Screening to detect alcohol abuse and counselling for adult population	12	60.0
Screening for domestic violence against women	11	55.0
Colorectal cancer screening annually for patients over 50 using faecal occult blood testing	10	50.0
Tobacco use screening and cessation counselling	9	45.0
Screening adults for depression	8	40.0
Screening postmenopausal women for osteoporosis	7	35.0
Vaccination for adults (MMR, Varicella, Pneumococcus, Influenza, Diphtheria)	6	30.0
Screening activities during periodic health check-up (second round)	n	Percent
Vaccination for HPV for both sexes between age 9–26 years	8	40.0
Screening on Road safety and counselling on seat-belt use, drinking and driving	11	55.0
Screening Mammography examination every 1–2 years for women over 40 years	17	85.0
Screening for Domestic violence against women	7	35.0
Colorectal cancer screening for patient over 50 with annual faecal occult blood testing	16	80.0

**Table 4:** Delphi first round, experts’ agreement on whether the IHS framework for causal relationships of diseases and the intervention approaches are plausible for the Ethiopian context (*n* = 20)

Experts agreement on IHS framework	n	Percent
IHS has appropriate interventions to manage common modifiable factors from causing immediate risk factors	20	100.0
IHS has an appropriate intervention to manage intermediate risk factors from causing main diseases	19	95.0
IHS has an appropriate intervention to manage main diseases from causing socioeconomic, cultural, and environmental and political problems	18	90.0
HS has an appropriate intervention to manage socio- economic, cultural, and environmental and political problems from leading to common modifiable risk factors	18	90.0
IHS framework for causal relationships of diseases and the intervention approaches are plausible	18	90.0

#### Case finding for hypertension and cardiovascular diseases

Despite the experts’ recommendation and the common expectation from routine medical practices that all health professionals measure patients’ vital signs prior to providing any treatment [31], the blood pressure of 47.2% (*n* = 394) of the patients had not been checked, indicating that case finding for hypertension was not optimally integrated in the routine health care system. However, the experts recommended making blood pressure measurement of all patients a routine medical practice by including it in the patient care guideline. To ensure all patients’ blood pressure is measured, the experts suggested that clinical audits be conducted regularly in all health facilities. Likewise, 90% (*n* = 18) of the experts reached consensus on making physical examination and laboratory tests for screening of cardiovascular diseases (such as coronary heart diseases) and assessment of patients over the age of 40, for first stroke risk, part of the patient care guidelines.

#### Case finding for diabetes

The case finding for diabetes at health facilities appeared very weak when 90.0% (*n* = 751) of the patients had not been asked if they had diabetes or symptoms of diabetes. The effect of such oversight was accentuated by 31.8% (*n* = 8) of the health facilities reporting diabetes mellitus as the most common prevalent chronic disease in their health facility. The experts (85%, *n* = 17) recommended assessing patients’ risks and screening for diabetes as a routine part of health examination or general physical examination. The experts pointed out that conducting blood sugar tests as a routine activity at clinics was easy and feasible, yet not widely practiced, especially for children. The experts suggested that screening priority be given to patients who had a high risk for diabetes, such as people with BMI > 25, a family history of diabetes or some genetic predisposition, people who lived unhealthy lifestyles, and patients with other chronic NCDs.

#### Case finding for the common female cancers (cervical and breast cancer)

The health professionals’ focus on cancer was limited as 91.9% (*n* = 793) of the patients had not been asked if they had any history of cancer nor were they advised on having a screening test for cancer. Of the experts, 90% (*n* = 18) agreed on applying case finding for cervical and 80% (*n* = 16) agreed for breast cancer. For female patients, PAP smear test for cervical cancer’s early diagnosis and treatment was not widely practiced at the health facilities because 92.21% (*n* = 296) of the patients had never been asked by the service providers during their contact if they had had a PAP test or were they advised on the importance of having the test. For 93.3% (*n* = 735) of the patients aged 18–25 years, HPV vaccination service or advice on its importance had not been provided which indicated loose case finding for cervical cancer and its prevention. Breast cancer was the second most prevalent of female cancers in Sub-Saharan Africa [32]. Yet, of the patients, 89% (*n* = 453) did not receive a simple clinical breast examination by a doctor, nurse, or other health professional nor were they advised on regular self-breast examination to detect breast lumps. Of the eligible female patients, 85.2% (*n* = 264) had never had a breast mammographic check-up. The health professionals did not emphasise breast cancer case finding, given that of the female patients over 40, 91.2% (*n* = 291) had not been asked if they had had a mammographic test for breast cancer.

#### Case finding for the common infectious diseases (HIV, TB & STI)

Considering the country’s set-up, 85% (*n* = 17) of the experts, agreed that case finding should be conducted for HIV/AIDS and 80% (*n* = 16) for TB. The case finding for HIV was widely compromised as 67.7% (*n* = 567) of the patients had not been asked about previous HIV test status nor initiated and counselled for HIV testing. The Ministry of Health (2010b:36) stipulates that all patients attending OPD are expected to be briefly counselled and encouraged to undergo HIV testing unless they refuse. Of the patients, 86.8% (*n* = 724) were not assessed for STI risk behaviour. Of the experts, 75% (*n* = 15) agreed that patients should be examined for STI prevention and treatment. Considering that 15.4% (*n* = 128) of the patients’ self-risk perception of HIV infection was assessed to be medium to high risk and 24.8% (*n* = 207) had never been tested for HIV infection, there is a great need for preventive care for STIs. In addition, 50% (*n* = 11) of the health facilities did not provide or actively engage in case finding and gave different reasons for not doing so. The health facilities indicated that they did not do case finding preventive care because of fear over patients’ willingness to accept the test; high patient flow; lack of laboratory facilities to conduct the tests; lack of awareness of the case finding approach, and their focus on patients’ complaints only.

### Periodic health examination

A periodic health examination refers to a general physical examination of patients, not an examination for a specific injury, illness, or condition, which should be provided for patients regularly within a specific period of time [9]. Of the health facilities, 63% (*n* = 14) did not provide periodic health examinations (PHE) while 77.9% (*n* = 651) of the patients had not been going for routine check-ups of their health (periodic medical check-ups). To improve such low performance and poor service utilization, all the experts (100%; *n* = 20) recommended making periodic health examinations available for patients who are apparently not sick(see Table 3). The experts agreed that the provision of preventive risk assessment services especially for patients with high risk factors, such as family history, obesity and hypertension, was important. However, considering the poor economic status that caused financial barriers to making PHE available for the community, the experts (95%; *n* = 19) advised including the periodic general health check-ups in community or individual insurance system.

The experts agreed that the following health interventions should be applied in annual PHEs: screening for hypertension and its complications (95%; *n* = 19); screening for diabetes in patients with hypertension or BMI > 25 (95%; *n* = 19); measuring blood cholesterol level and counselling on healthy diet and obesity (85%; *n* = 17); Pap smear screening every 3 years beginning from age 21 (85%; *n* = 17); clinical breast examination every 1–2 years for women over 50 years (80%; *n* = 16); STI screening and counselling (80%; *n* = 16); visual acuity screening, using the Snellen sight chart (80%; *n* = 16); counselling on physical activity (75%; *n* = 15); screening by mammography examination of women over 40 years every 1–2 years (85%; *n* = 17), and screening for colorectal cancer for patients over 50 years using faecal occult blood testing (80%; *n* = 16).

It is important for health care workers to have a guide or checklist on daily prevention and preventive practices that are part of the PHE. Of the experts, 90%(*n* = 18) recommended that guidelines and protocols be prepared on conditions that must be sought and monitored during check-ups; periodic health check-ups be linked with the insurance system; health facilities start allocating funds for promotive disease prevention purposes; patients who have positive results upon screening and who cannot afford the treatment should have health insurance support for their specific health problem, and health facilities and health professionals should be adequately prepared in terms of staff numbers and competencies, and equipment and supplies to provide such promotive and preventive activities. The experts (90%; *n* = 18) agreed that the implementation of periodic health check-ups should not be applied in the form of a mandatory service, but patients should be encouraged to make use of the opportunity, and assessing the family, domestic and social background of a patient should be incorporated in the check-up process. In addition, the experts pointed out that the community/country’s disease burden identification needs to be conducted regularly to update the disease lists upon which periodic check-ups are based, and the shortage of capable health education and promotion specialists that coordinate, plan and implement such interventions must be improved.

### Experts agreement on IHS framework

The experts were asked to indicate whether the Integrated Health Service (IHS) framework applied in the study was practical. The IHS framework, adapted by the researcher from the linear “causation framework”, was proposed in the study to address the relationships among predisposing factors and diseases together with the type of interventions needed in each step (IHS framework in Fig. 1 was provided to the experts for their review).

#### IHS disease causal relationship

The experts’ consensus was sought on interventions in each step of an integrated health system as an effective approach to curb the predisposing factors causing health problems. Accordingly, the experts were asked if an integrated approach in health promotion, disease prevention and curative care (as indicated in Fig. 1) encompasses the appropriate interventions to comprehensively manage *common modifiable factors* (e.g., tobacco and alcohol use) from causing immediate risk factors (e.g., raised BP). The experts (100%; *n* = 20) agreed unanimously that the IHS intervention approach is appropriate to curb progression of common modifiable factors that cause immediate risk factors (see Table 4). The experts’ consensus gave approval to the researcher’s initial notion that each intervention in the IHS framework needs to have an integrated health promotion, disease prevention and curative approach in order to break the progression to the next step, and thus achieve a lasting effect.

The experts were also asked if an integrated health service approach (health promotion, disease prevention and curative care) was the appropriate intervention to manage *intermediate risk factors* (e.g., raised BP) from causing main diseases (e.g., heart failure). Of the experts, 95% (*n* = 19) agreed that the proposed IHS framework stipulates an appropriate intervention mechanism to manage intermediate risk factors from causing main diseases(Table 4). The consensus was very useful and in line with the researcher’s premise that interventions are comprehensive when integrated as promotive, preventive and curative aspects in order to halt the progression of immediate risk factors to full blown diseases.

The experts’ consensus was sought on whether an integrated approach (health promotion, disease prevention and curative care) was the appropriate intervention to manage *common main diseases* (heart disease, stroke, cancer) from causing socio-economic, cultural, environmental and political problems (e.g., poverty). Of the experts, 90% (*n* = 18) agreed that common main diseases led to socioeconomic, cultural, and environmental and political problems. The experts’ approval was crucial in that it is not uncommon for health professionals to overlook health promotion and disease prevention as an essential patient management component once patients develop main diseases. In addition, the experts’ consensus validated the premise that a population suffering heavily from the main non communicable diseases can lead to the social determinant of health, such as poverty. This completed the IHS framework premise that the vicious cycle continues until intervention is holistically applied in an integrated manner. According to the WHO [33], there is a vicious cycle that interconnects poverty and chronic disease. The UN General Assembly [34] declared that the prevention of chronic diseases is the cornerstone in breaking the vicious cycle by which poverty, chronic diseases and other risk factors fed off each other, creating a deadly spiral of sickness and deprivation.

The experts were asked whether an integrated approach (health promotion, disease prevention and curative care) was the appropriate intervention to manage socio-economic, cultural, environmental and political problems (e.g., poverty) from leading in to common modifiable risk factors (e.g., tobacco and alcohol use). Of the experts, 90% (*n* = 18) agreed while 10% (*n* = 2) remained neutral (see Table 4). The experts’ consensus substantiated a wider perspective on the integrated approach as an effective way to halt the predisposition of social determinants of health, such as population growth, low income, population aging, and environmental hygiene, to common modifiable risk factors.

#### IHS framework interventions approach

The experts were asked whether the IHS framework for causal relationship of diseases and the intervention approaches was plausible for the country context. Of the experts, 90% (*n* = 18) agreed that the IHS framework was plausible while 10% (*n* = 2) remained neutral. The strong positive response by the experts indicating their approval of this framework, authenticates its applicability for chronic non-communicable, communicable, and nutrition-related diseases in the Ethiopian set-up and possibly applicable for other LMIS countries as well.

## Discussion

A primary health care approach for addressing NCDs encourages long-term investment in prevention-focused health care systems and emphasizes responses which prioritize cost-effective, primary care-based interventions [35]. The IHS strategy, which aligns with the country health policy [36], is applicable for existing PHC services with an efficient and locally feasible approach. It entails developing and revising the existing health care protocols in such a way that both promotive and preventive health care is included in the health care system. A culture of providing comprehensive health care has to be developed instead of only focusing on specific disease treatment. According to Maher et al. [37], interconnected delivery of preventive and curative health care is important as providing curative care without prevention and vice versa represents missed opportunities for both modalities. Providing treatment and avoiding patients’ promotive and preventive health care needs results in incomplete health care. In order to address patients’ health needs comprehensively, there is a need for a conceptual framework that indicates the relationships among predisposing factors and diseases, and which guides the type of intervention required from health professionals at specific stages. The Integrated Health Service (IHS) framework was presented to the experts for consideration as a means of addressing the health facilities’ omissions in terms of providing comprehensive health care to their patients [16].

The experts suggested that case finding for both hypertension and cervical cancer could plausibly be sought for patients who attended medical care in health facilities. Consensus was also reached on having case finding for hypertension, diabetes, cervical cancer, breast cancer, HIV, TB, and for patients at high risk for STI. The experts did not agree on having case finding screening for obesity, smokers, physical inactivity, chronic obstructive pulmonary disease, and harmful use of alcohol. The experts were agreed on conducting periodic health examination for patients who are apparently not sick and advised to include the periodic health check-ups in the community or individual insurance system. The experts’ recommended that focal areas for periodic health check-ups should be screening for hypertension and its complications; screening for diabetes for patients with hypertension or BMI > 25; measuring blood cholesterol level and counselling on healthy diet and obesity; Pap smear screening every 3 years beginning at age 21; clinical breast examination every 1–2 years for women over 50 years; STI screening and counselling; visual acuity screening using Snellen sight chart, and counselling on physical activity.

The experts accepted the Integrated Health Service (IHS) framework as a comprehensive framework which indicates the cause-and-effect of chronic disease progression and the appropriate interventions to apply. Asked whether the Integrated Health Service (IHS) framework for causal relationships of diseases and the intervention approaches was plausible for the Ethiopian context, the majority (90%; *n* = 18) of the experts agreed, substantiating the IHS framework’s applicability for chronic non-communicable, communicable, and nutrition-related diseases. Moreover, the experts agreed unanimously (100%; *n* = 20) that an IHS approach in health promotion, disease prevention and curative care was the appropriate instrument to manage common modifiable factors (e.g., tobacco and alcohol use), thus preventing them from causing intermediate risk factors (e.g., raised BP).

Health staff should make use of the opportunity when people are seen in primary care to identify and address modifiable risk factors; screen for common NCDs, and diagnose, treat and follow-up patients with common NCDs, using standard protocols [37]. Making use of patients’ presence at health facilities for promotive and preventive health care purposes is justifiable in the Ethiopian context for several reasons. Firstly, from phase 1 study, of the patients, 88.8% (*n* = 739) frequently visited health facilities for treatment of acute or chronic illnesses, therefore, it will be wise to recognise their promotive and preventive health needs when they come for curative health care. For example, of the patients, 96.5% (*n* = 796) indicated that they had not been asked or tested for cholesterol while 2.0% (*n* = 17) indicated that their blood cholesterol level was tested while attending the health facility. This indicates that the opportunity for preventive cholesterol level screening of those patients could have been lost if the health professionals had not enquired about previous testing.

The practice of building a healthy lifestyle, such as by making exercise a routine habit, practising healthy eating habits, and avoiding the harmful use of alcohol and cigarette smoking, is not widely adopted in the population culture. In this regard, 95% (*n* = 19) of the experts advised that health professionals should routinely link health promotion and disease prevention to curative service (such as advice on healthy diet, physical exercise and so on). Therefore, for the health professionals, patient contact would be a golden opportunity to provide support through educating and counselling on a healthy lifestyle. Of the experts, 95% (n = 19) agreed that there was a need to reorient the current health promotion and disease prevention approach in such a way that the monitoring system is strengthened and the health professionals’ responsibility and accountability at all levels are ensured. By doing so, health professionals’ sole focus on the treatment aspect of patient care instead of providing an integrated health care service will change over time and a better understanding of the aims of an integrated healthcare service by means of effective in-service training will lead to optimal patient care.

Of the experts, 95% (*n* = 19) further agreed that the IHS was an appropriate intervention framework to manage intermediate risk factors by means of an integrated promotive, preventive and curative process thus preventing the progression of intermediate risk factors to full-blown diseases. The experts (90%; *n* = 18) also reached consensus on the need for much broader interventions on effective ways to halt the predisposition of social determinants of health, such as population growth, low income, population aging, and environmental hygiene, to common modifiable risk factors such as harmful tobacco and alcohol use [20]. The experts (90%; *n* = 18) acknowledged the IHS framework as an ideal approach to comprehensively address the health needs of the population in the Ethiopian context.

Promotive and preventive health care are more cost effective than that of curative treatment and thus the health-related costs of underuse of recommended clinical preventive services are substantial. Increasing the use of selected (nine) clinical preventive services to more optimal levels (i.e., levels achieved by high-performing health plans) could prevent an estimated 50,000–100,000 deaths each year among adults aged < 80 years [38]. The Ethiopian Health sector transformation plan, 2015/2016–2019/2020 [39] gives high priority to prevention health care, but the findings from phase 1 of this study indicated that the government’s quest for delivering comprehensive health care has not yet been achieved at the functional level of health facilities [16]. The country’s current health care cost for treatment can be decreased considerably and patient deaths can be prevented if the integrated health care approach is successfully applied.

## Conclusion

The Integrated Health Service (IHS) framework, adapted by the researcher from the linear “causation framework”, proposed in this study was agreed by experts to be a plausible method in describing the causal relationship of chronic non-communicable, communicable, and nutrition-related diseases. The IHS framework can be an efficient and effective intervention approach in dealing with patients who are at risk for diseases, by encouraging a healthy lifestyle, preventing disease occurrence, and preventing disease progression and improve quality of life in patients who are already sick. Therefore, the framework can play a vital role for the country, possibly for other LMICs as well, in decreasing the overall disease burden by preventing the acquiring, progression, suffering or dying from diseases through restraining the vicious cycle of chronic diseases.

## Additional files

Additional file 1: Delphi first round questionnaire (DOCX 327 kb)

Additional file 2: Delphi second round questionnaire (DOCX 21 kb)

## Data Availability

The datasets used and/or analyzed during the current study are available from the corresponding author on reasonable request.

## Abbreviations

AIDS: Acquired Immunodeficiency Virus
CDC: Center for Disease Control and Prevention
HBV: Hepatitis B Virus
HIV: Human Immunodeficiency Virus
HPV: Human Papilloma Virus
ICSI: Institute for Clinical Systems Improvement
IHS: Integrated Health Service
LMICs: Low and Middle Income Countries
PAP: Papanicolaou
PHC: Primary Health Care
STIs: Sexually Transmitted Infections
TB: Tuberculosis
UNISA: University of South Africa
USPSTF: US Preventive Services Task Force
WHO: World Health Organization
