# Supplementary material for: The integrated health service model: the approach to restrain the vicious cycle to chronic diseases
Source: BMC Health Serv Res. 2019 May 31;19:347. doi: 10.1186/s12913-019-4179-x (PMC6544908; doi:10.1186/s12913-019-4179-x)
Supplement: Supplementary file 1 — Delphi first round questionnaire (DOCX 327 kb) [file 12913_2019_4179_MOESM1_ESM.docx]

### Delphi questionnaire round 1 consent

Dear Sir/Madam

Thank you for accepting my invitation to read through the online consent form and to be a candidate for my research project.

I, Netsanet Fetene, am a Doctoral (DLitt et Phil) student at UNISA in the Department of Health Studies. I am conducting a study on the integration of health promotion and disease prevention services into the curative health care being provided at Addis Ababa hospitals and health centres. The study is entitled “Integration of promotive, preventive and curative health care services in public hospitals and health centres of Addis Ababa, Ethiopia”.

This second phase of the study seeks the participation of experts who have worked and have experience in health promotion, disease prevention and treatment services particularly in government and non-governmental organisations. It is understood that experienced programme leaders, project officers, government officials and service providers constitute a significant proportion of the health care workforce playing a considerable role in making recommendations pertaining to health promotion, disease prevention and curative health care integration in the Ethiopian context.

The goal of the study is to explore the level of health promotion and disease prevention services provided at hospitals and health centres. The study also investigates what factors prohibit the provision of these services so that recommendations can be made for service improvements. The first phase of the study involved 836 patients and the health service managers of 22 health facilities and exit interviews conducted in the 22 health facilities. The second phase of the study, which pertains to this request, applies the Delphi consensus seeking technique, using medical experts to draw realistic conclusions and recommendations based on the findings of phase 1 of the study.

The study has the following inclusion criteria which I believe you duly fulfil. These inclusion criteria for your profession are as follows:

- Must have 10 or more years’ work experience in the Ethiopian health service provision or health programme management.
- Must be currently working in Addis Ababa.
- Willing to participate.

This research will be carried out using the Delphi technique consisting of three questionnaires (known as rounds) aiming to achieve consensus. Simple, specific questions are posed, supported by background findings from phase 1 seeking your level of agreement. The amount of time necessary for completion of the questionnaire in Round 1 will vary with each panellist as it has mixed Likert scale and open-ended questions, but round 2 and 3 should not take more than 30-40 minutes. There are no right or wrong answers to the questions. This study is seeking your expert opinion. I hope you will find the process interesting and results will be made available to you at the conclusion of this study.

It is important that you understand that your participation in this study is entirely voluntary. If you do not wish to take part in this study, it will not affect you in any way. In addition, any information that you provide will be confidential and when the results of the study are reported, you will not be identified in the findings. Your name will not be recorded in any rounds; instead you will be allocated a unique code that is only identifiable to the researcher. You will remain anonymous to the other participants (20 experts have been invited) throughout this Delphi study and only the researchers will be able to identify your specific answers. Return of completed Delphi rounds implies consent to participate in this study. If you meet the inclusion criteria and would be willing to participate in the study, I would be grateful if you could complete the enclosed questionnaire and return it at your earliest convenience (preferably within one week’s time) to me using online form, OR my personal mail address netsanetfetene@gmail.com, OR drop the filled hard copy in an envelope at Yale Office, Near protection Building, Pizza corner, 4th floor, Chechenia, Addis Abba. I sincerely hope you will agree to participate. If you have any questions, please e-mail Dr Netsanet Fetene, netsanetfetene@gmail.com or call +251910137506.

Thank you for your time and any help you may be able to offer to this study. If you consent to participate in the study, please proceed to complete the questionnaire.

Yours sincerely

Netsanet Fetene (MD, MPH)

**UNISA, PHD (DLitt et Phil) Candidate**

###

### Delphi consensus seeking questionnaire round 1

Please answer the following 35 questions by indicating your level of agreement, or by providing an open response where indicated, after you have read the background information based on the findings of phase 1 of this study. Your answer should be based on the Ethiopian context.

1. Do you agree that periodic health examinations for healthy individuals for general health check-ups or certain diseases screening is recommended?

Background

A periodic health examination is a general physical examination of patients, not an examination for a specific injury, illness, or condition which is to be provided for patients in a regular specific period of time (for example: every 1-2 year). Findings from phase1: Two-thirds of the health facilities (n=14; 63%) did not provide periodic health examinations. Asked of their previous experience, 77.9% (n=651) of the patients had not been for periodic check-ups of their health (periodic medical check-ups).

- Strongly agree
- Agree
- Neutral
- Disagree
- Strongly disagree

2. If you agreed or strongly agreed with question 1, in recommending general health check-ups, would you advise that general health check-ups be covered by the community (which applies through government system) or individual insurance systems?

- Strongly agree
- Agree
- Neutral
- Disagree
- Strongly disagree

3. If you agreed or strongly agreed with question 1 in recommending general health check-ups, mark the periodic check-ups that can feasibly be conducted.

- Tobacco use screening and cessation counselling
- Measuring blood cholesterol level and counselling on healthy diet and obesity
- Screening for hypertension and its complications
- Counselling on physical activity
- Screening for Diabetes in patients with hypertension or a BMI>25
- Annual colorectal cancer screening for patients over 50 by means of faecal occult blood testing
- Clinical breast examination every 1-2 years for women over 50 years
- Mammography examination every 1-2 years for women over 40 years
- Pap smear screening every 3 years beginning at age 21
- Vaccination for adults (MMR, Varicella, Pneumococcus, Influenza, Diphtheria),
- Vaccination for HPV for both sexes between ages 9-26 years
- STI screening and counselling
- Screening on road safety and counselling on seat-belt use, drinking and driving
- Visual acuity screening using Snellen’s sight chart
- Screening postmenopausal women for osteoporosis
- Screening to detect alcohol abuse and counselling for adult population
- Screening for domestic violence against women
- Screening adults for depression
- Other:

3.1 Any personal recommendations, comments or experience on routine health check-ups you would like to share? ------------------------------------------------------------------------------

4. Would you recommend that case finding for certain diseases take place in health facilities?

Background

Case finding is testing or screening of patients for another condition other than the one for which they sought medical care. Half of the health facilities (n=11; 50%) do not provide case finding preventive services. The major reasons given by the health facilities that did not provide case finding preventive services were fear of patients’ willingness to accept the test; high patient flow; lack of laboratory instruments to conduct the tests; service providers’ lack of awareness of case finding approach, and their focus on patients’ complaints only.

- Strongly agree
- Agree
- Neutral
- Disagree
- Strongly disagree

5. If you agreed or strongly agreed on having case finding testing in question 4, please mark the case finding testing that can feasibly be conducted. Listed below are some possible cases, please include any cases not listed that you feel important to detect in case findings in “Other”.

- Hypertension and related complications
- COPD (Chronic Obstructive Pulmonary Disease)
- Diabetes
- Cervical cancer
- Obesity
- TB
- HIV
- Physical inactivity
- Smokers
- Harmful alcohol users
- Other:

5.1 Any personal recommendation, comment or experience on case finding test you would like to share? -----------------------------------------------------------------------------------------

6. Do you agree that measuring the blood pressure of all patients should be included in the patient care guidelines?

Background

Findings from phase 1 showed that most of the health facilities (86.4%; n=19) had adequate blood pressure apparatus for each examination room. All the health facilities (100%; n=22) had sufficient skilled staff who can take blood pressure, and most of them (90.9%; n=20) had adequate anti-hypertensive drugs to treat their patients. However, the blood pressure of almost half (n=394; 47.2%) of the patients was not checked during their recent visit to the health care facility.

- Strongly agree
- Agree
- Neutral
- Disagree
- Strongly disagree

7. Knowledge and skills of hypertension prevention approaches, such as advice on lifestyle (e.g., on smoking and stress), healthy diet and physical exercise, were lacking in the majority of health service providers working in the selected hospitals and health centres.

- Strongly agree
- Agree
- Neutral
- Disagree
- Strongly disagree

8. Do you agree that Ethiopian health facilities (hospitals and health centres) need to initiate a health care unit comprised of health professionals specialized in the provision of health promotion, disease prevention and rehabilitation services pertaining to patients with non-communicable diseases, including hypertension?

- Strongly agree
- Agree
- Neutral
- Disagree
- Strongly disagree

9. Despite the fact that sufficient skilled staff and adequate BP apparatus for measuring blood pressure were available, a high percentage of the patients whose blood pressure was not measured, was high. What recommendations can you suggest to improve this situation?

Background information: In Ethiopia, 51% of deaths are attributed to non-communicable diseases, of which the leading cause of death was cardiovascular disease (24%), followed by hypertension (12%) and stroke (11%) (Misganaw et al 2012).

10. The majority of the health service providers working at the selected hospitals and health centres lacked awareness, knowledge or skills pertaining to the prevention and treatment of hyperlipidaemia (elevated blood cholesterol level), such as providing advice on lifestyle, healthy diet, and physical exercise and treating patients suffering from the diseases.

Background

Findings from phase 1 showed that 81.1% (n=678) of the respondents attending the health facilities indicated that their blood cholesterol level had never been checked. Of the health facilities, 68% (n=15) did not have the equipment to measure blood cholesterol level, medical doctors who could diagnose and treat raised blood cholesterol, or the essential medication to decrease raised cholesterol levels. Of the health facilities, 81.8% (n=18) did not have enough health education or promotion resource materials for their patients.

- Strongly agree
- Agree
- Neutral
- Disagree
- Strongly disagree

11. There are guidelines that emphasize the importance of having a fasting cholesterol test done every five years beginning at age 20 (Harvard Medical School 2009:3). Do you agree that the measuring of blood cholesterol levels of all patients (those who have never been tested and those eligible for repeated cholesterol testing) should be included in the patient care guidelines?

- Strongly agree
- Agree
- Neutral
- Disagree
- Strongly disagree

12. What recommendations would you make to improve the health facilities’ low performance in terms of health promotion, prevention and effective treatment of hyperlipidaemia (elevated blood cholesterol level)? ------------------------------------------------

13. Should physical examination and laboratory tests for screening of cardiovascular diseases (such as coronary heart diseases) be included in the patient care guideline?

Background

Cardiovascular diseases (CVDs) are a group of disorders of the heart and blood vessels that are the number one cause of death globally (WHO 2015b). Of the patients interviewed, only 2.9% (n=24) were asked if they had a history of chest pain that radiated down the arm, which indicated the low emphasis of the service providers in taking history on important symptoms of cardiovascular diseases. Of the health facilities, only 22.7% (n=5) had doctors or internists who could diagnose and treat cardiac-related conditions, and 31.8% (n=7) had the proper medication to treat cardiovascular diseases. The health facilities did not have the necessary equipment to diagnose coronary heart disease, except for 13.6% (n=3) which had EKG equipment.

- Strongly agree
- Agree
- Neutral
- Disagree
- Strongly disagree

14. Based on the background information provided about the health facilities’ readiness for the prevention and treatment of cardiovascular diseases, what recommendations would you make to improve the prevention and treatment of cardiovascular diseases in the Ethiopian context?

15. Should it be recommended for the Ethiopia context that patients over a certain age (40 years) undergo patient assessment for first stroke risk?

Background

It is helpful for patients if healthcare providers are able to estimate a person’s risk for a first stroke. According to the American Heart Association and American Stroke Association (2011:562) each patient with stroke risk, such as age over 40, hypertension, diabetes, cardiac diseases, obesity, cigarette smoking, or being male, should undergo an assessment of stroke risk. Of the respondents aged over 40 years, only 7% (n=19) were asked if they had a history of having a stroke, or were assessed and advised on the risk of the disease.

- Strongly agree
- Agree
- Neutral
- Disagree
- Strongly disagree

16. Would you agree that it is advisable to counsel patients on cancer predisposing factors and use the opportunity of face-to-face patient contact during attendance at health facilities to advise patients and do specific cancer screening, where indicated?

Background

The findings from phase 1 showed that of the patients, 91.9% (n=793) were not asked if they had any history of cancer nor were they advised on having a screening test for cancer

- Strongly agree
- Agree
- Neutral
- Disagree
- Strongly disagree

16.1 Could you make recommendations, provide comments or share experience with regard to cancer screening? ------------------------------------------------------------------------------

17. Do you agree that visual acuity testing using Snellen's chart needs to be part of a routine eye examination during a general physical examination?

Background

Findings from phase 1 showed that of the patients, 89.6% (n=742) were not examined for visual impairment.

- Strongly agree
- Agree
- Neutral
- Disagree
- Strongly disagree

18. Do you agree that patient screening for diabetes should be part of a routine health examination or general physical examination?

Background

The first phase found that of the respondents, 90.0% (n=751) were not asked if they had diabetes or symptoms of diabetes. According to the International Diabetes Federation (IDF) (2014), Ethiopia is one of the top three countries in Africa with a 4.48 diabetes prevalence rate, 2.1 million people living with diabetes and 34,262 deaths occur each year from diabetes and the complication related diabetes.

- Strongly agree
- Agree
- Neutral
- Disagree
- Strongly disagree

18.1 Could you make any recommendations, provide comments or share your experience on diabetes screening? ---------------------------------------------------------------------------------------------------------------------------------------------------------------------------------------------------------

19. Do you agree that enquiring about a patient’s smoking status and providing relevant advice should be an essential component of health care in health centres and hospitals to play a key role in fighting tobacco use?

Background

The first phase found that 94.7% (n=788) of the patients were not asked if they smoked cigarettes. According to the CDC (2015a), health professionals can play a key role in fighting tobacco use because for smokers who want to quit, getting started often needs support and motivation from trusted sources like health service providers.

- Strongly agree
- Agree
- Neutral
- Disagree
- Strongly disagree

20. Would you agree that a rehabilitation unit for diseases related to cigarette smoking addiction should be integrated into the health care system?

Background

Phase 1 found that none of the health facilities (0.0%; n=0) had an addiction rehabilitation unit nor did they conduct laboratory tests to monitor blood nicotine levels for the patients who smoked cigarette and wanted to quit smoking. Integrating the rehabilitation unit for diseases related to addiction such as cigarette smoking, in the health care system is essential for the identification of smokers, effective treatment and helpline referral for quitting coaching (Leuthard et al 2015:65).

- Strongly agree
- Agree
- Neutral
- Disagree
- Strongly disagree

20.1 Can you make any recommendations, provide comments or share your experience on the prevention of cigarette use and addiction? --------------------------------------------------

21. Do you agree that screening for harmful use of alcohol, relevant counselling and referral for treatment should be integrated in routine healthcare provision?

Background

The first phase found that of the patients, 92.3% (n=770) were not asked about their alcohol consumption by the service providers. Talking with patients about their drinking is the first step of screening and brief counselling (CDC 2014a). The WHO (2014b:65) emphasises that health services play a critical role in tackling alcohol-attributable harm by screening and intervention with referral for treatment (SBIRT), which has been found both effective and cost-effective in a variety of health service settings.

- Strongly agree
- Agree
- Neutral
- Disagree
- Strongly disagree

22. Do you agree that rehabilitation units for the diagnosis and treatment of harmful use of alcohol should be available at health facilities?

Background

Phase 1 found that none of the health facilities (0.0%; n=0) had laboratory facilities to measure blood alcohol levels or a rehabilitation unit to help quit alcoholism. The WHO (2014b:289-305) reported that the Alcohol per capita (APC) rate for Ethiopia was 4.2% with 3.1% of Ethiopian drinkers being heavy episodic drinkers (HED).

- Strongly agree
- Agree
- Neutral
- Disagree
- Strongly disagree

22.1. Can you make any recommendations, provide comments or share your experience on the harmful use of alcohol? ------------------------------------------------------------

23. Do you agree that screening of patients’ nutritional status needs to be assessed and reported, and that healthcare staff need to recognize this as an important aspect of patient care?

Background

Phase 1 found that 79.7% (n=665) of the patients were not assessed on their nutritional status or advised or educated on nutrition, and 50% (n=11) of the health facilities did not have staff who could diagnose and treat diseases related to poor nutrition.

- Strongly agree
- Agree
- Neutral
- Disagree
- Strongly disagree

24. Do you agree that routine health care needs to include patients’ Body Mass Index (BMI) assessment in order to evaluate disease risk and diagnose disease status related to obesity or underweight?

Background

Phase 1 indicated that of the patients, 82.4% (n=688) had not had their body weight measured. Of the patients who reported that their weight and height was measured and their BMI calculated, 17.0% (n=55) were in the category of overweight, and 3.4% (n=11) were obese. A BMI at or above the current cut point for overweight (BMI ≥25 < 29.9 kg/m2) is associated with fatal CHD and diabetes while a BMI at or above the current cut point for obesity (BMI ≥30 kg/m2) is associated with an elevated risk for all-cause mortality compared with normal weight (18.5 to 24.9 kg/m2) (U.S. Department of Health and Human Services 2014b:39-41).

- Strongly agree
- Agree
- Neutral
- Disagree
- Strongly disagree

25. Do you agree that routine health care needs to include the assessment of patients’ level of physical exercise in order to advise on the importance of physical activity to decrease disease risks and treat diseases related to physical inactivity?

Background

Of the patients, 80.4% (n=671) were neither asked about their physical exercise habits nor advised on the importance of regular physical exercise. Of the patients who were asked if they had participated in any physical activities or exercises, such as running, calisthenics, or walking for exercise in the past month in order to keep themselves healthy, 83.8% (n=699) did not perform any of the noted physical exercises. Advising physical activity (PA) in the right “dosage” is a highly effective prescription for the prevention, treatment and management of the most common chronic health conditions encountered in clinical practice (CDC 2015:2).

- Strongly agree
- Agree
- Neutral
- Disagree
- Strongly disagree

26. Do you agree that health facilities need to be equipped with physical exercise and rehabilitation unit equipment for patients who require physiotherapy or exercise?

Background

Of the health facilities, only 4.5 % (n=1) had equipment for physical exercise and/or rehabilitation for patients who required physiotherapy or exercise.

- Strongly agree
- Agree
- Neutral
- Disagree
- Strongly disagree

26.1 Any personal recommendation, comment or experience on exercise to share? ---------------------------------------------------------------------------------------------------------------------------

27. In the Ethiopian context, selected adult immunization services, such as for HPV, influenza, and meningitis, need to be available at health facilities.

Phase 1 found that of the health facilities, 0.0% (n=0) provided adult immunization for influenza and male human papilloma virus (HPV), and only 13.6% (n=3) provided adult immunization for female HPV and for bacterial meningitis.

- Strongly agree
- Agree
- Neutral
- Disagree
- Strongly disagree

28. The Integrated Health Service (IHS) framework for causal relationship of diseases and the intervention approaches is plausible for the Ethiopian context.

The Integrated Health Service (IHS) framework , modified by the researcher from the linear “causation framework” , is proposed in this study to address the relationships among predisposing factors and diseases along with the type of interventions needed in each step (see Figure below).

- Strongly agree
- Agree
- Neutral
- Disagree
- Strongly disagree


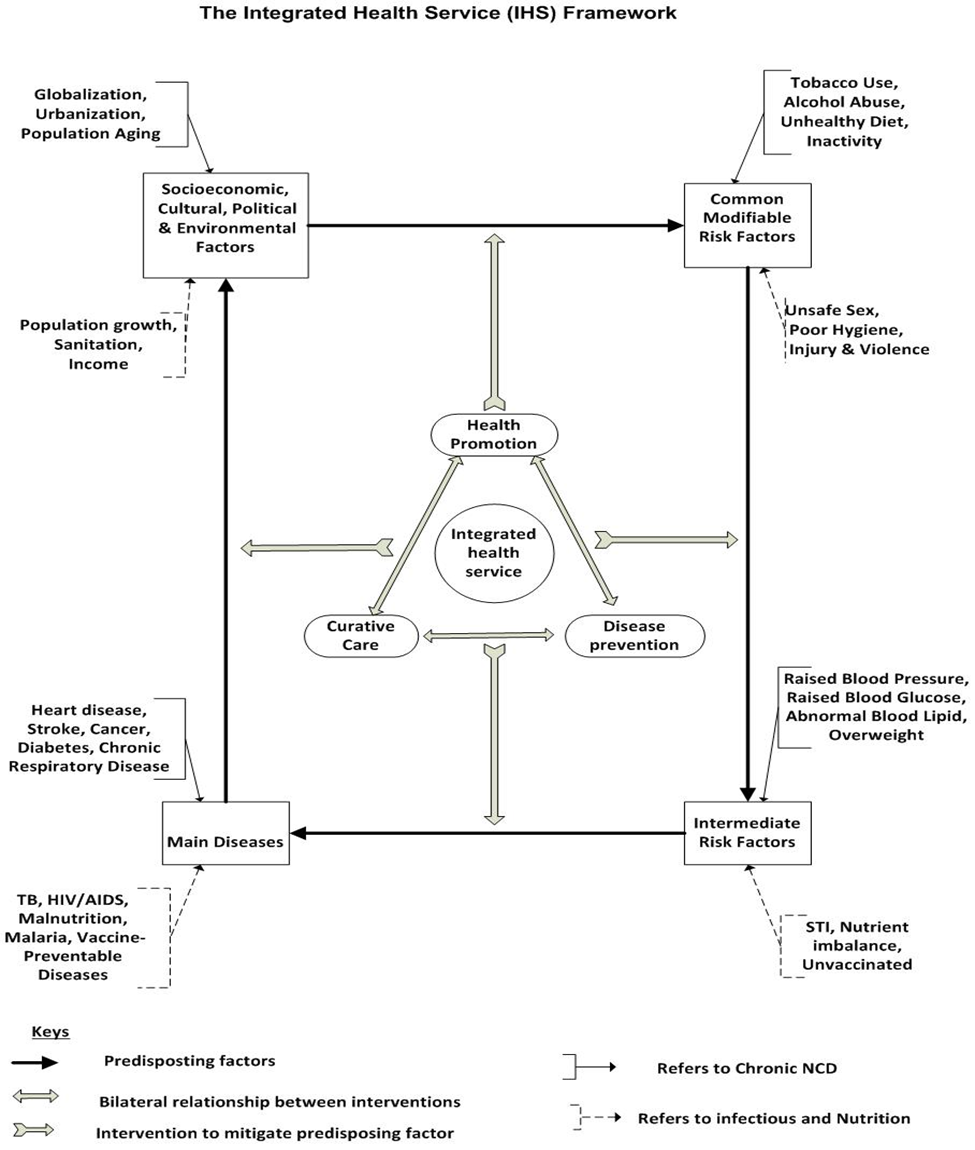


29. An integrated approach (health promotion, disease prevention and curative care) is the appropriate intervention to manage common modifiable factors (e.g., tobacco and alcohol use) from causing immediate risk factors (e.g., raised BP).

- Strongly agree
- Agree
- Neutral
- Disagree
- Strongly disagree

30. An Integrated approach (health promotion, disease prevention and curative care) is the appropriate intervention to manage immediate risk factors (e.g., raised BP) from causing main diseases (e.g. heart failure).

- Strongly agree
- Agree
- Neutral
- Disagree
- Strongly disagree

31. An integrated approach (health promotion, disease prevention and curative care) is the appropriate intervention to manage common main diseases (heart disease, stroke, cancer) from causing socioeconomic, cultural, environmental and political problems (e.g., poverty).

- Strongly agree
- Agree
- Neutral
- Disagree
- Strongly disagree

32. An integrated approach (health promotion, disease prevention and curative care) is the appropriate intervention to manage socioeconomic, cultural, environmental and political problems (e.g., poverty) from leading to common modifiable factors (e.g., tobacco and alcohol use)

- Strongly agree
- Agree
- Neutral
- Disagree
- Strongly disagree

33. Any personal recommendation, comment or experience regarding the approach of the IHS model you would like to share? ------------------------------------------------------------

34. Please write any final comment you think useful and worth noting in this round of the study ---------------------------------------------------------------------------------------------------------

35. Knowing your background, educational and work experience is very important for the study, please provide this information below

- Age
- Sex
- Highest education level
- Title of current position
- Work experience
- Total years of experience in health promotion and disease prevention

Thank you for completing the questionnaire.
