# Supplementary material for: The integrated health service model: the approach to restrain the vicious cycle to chronic diseases
Source: BMC Health Serv Res. 2019 May 31;19:347. doi: 10.1186/s12913-019-4179-x (PMC6544908; doi:10.1186/s12913-019-4179-x)
Supplement: Supplementary file 2 — Delphi second round questionnaire (DOCX 21 kb) [file 12913_2019_4179_MOESM2_ESM.docx]

### Delphi round 2 consent

Dear Sir/Madam,

Thank you for participating in the first round discussion and accepting my invitation to participate in this second round of Delphi consensus seeking discussion.

As I introduced myself in round 1 discussion, I, Netsanet Fetene, am a Doctoral (DLitt et Phil) student at UNISA in the Department of Health Studies. I am conducting a research study on health promotion and disease prevention services integration to the curative health care being provided at Addis Ababa hospitals and health centres. The study is entitled “Integration of promotive, preventive and curative health care services in public hospitals and health centres of Addis Ababa, Ethiopia”.

The goal of the study was to explore the level of health promotion and disease prevention services provided at hospitals and health centres. The study also investigated what factors prohibit the provision of these services so that recommendations could be made for service improvements. The first phase of the study involved 836 patient exit interviews conducted in 22 health facilities and the health service managers working at these facilities. The second phase of the study, which is the application of the Delphi technique, tries to draw recommendations through consensus seeking among the experts, using the findings from phase 1.

The first round of Delphi discussion was conducted with 20 experts, consisting of public health experts, health professionals working at different levels of the health sector, and health programme and policy makers. These experts were purposely selected to participate because the researcher believed they had rich experience (more than 10 years’ work experience) and would be able to provide variable perspectives on health promotion, disease prevention and curative health care in the Ethiopian context and were currently living in Addis Ababa. Please read the complete first round analysis and report available at the link below.

<https://drive.google.com/folderview?id=0B0qTnh9saQZSTUdyemFxNEN3dk0&usp=sharing>

Questions on which over 50% of the experts agreed during the first-round discussion but did not reach the consensus cut-off point (75%) as well as recommendations that some experts provided but that require other experts’ agreement, are forwarded to this second-round discussion. The second-round discussion has only 13 Likert scale questions in check-box format. It should take only 10-15 minutes of your time to complete. As experts reached consensus earlier than expected, this round will be the last discussion.

It is important that you understand that your participation in this study is entirely voluntary. If you do not wish to take part, it will not affect you in any way. In addition, any information that you provide will be confidential and when the results of the study are reported, you will not be identified in the findings. Your name will not be recorded in any rounds. Instead, you will be allocated a unique code that is only identifiable to the researcher. You will remain anonymous to the other participants (the invited 20 experts) throughout this Delphi study and only the researcher will be able to identify your specific answers. Returning the complete questionnaire is considered consent to participate in the study. I sincerely hope you will agree to participate. If you have any questions please e-mail Dr Netsanet Fetene, netsanetfetene@gmail.com or call +251910137506.

Thank you for your time and your help offered in this study.

Yours sincerely

Netsanet Fetene (MD, MPH)

**UNISA, PHD (DLitt et Phil) Candidate**

### Delphi consensus seeking round 2 discussion questionnaire

1. Please mark only the areas you still think important to have periodic health check-ups in the Ethiopian context (you may not necessarily agree with any of the options).

A periodic health examination is a general physical examination of patients, not an examination for a specific injury, illness, or condition, which is to be provided for patients at a regular specific period of time (for example, every 1-2 years). During the first round more than 50% expert agreement was reached but not the consensus cut-off point (75%) on the following periodic health check-ups:

- Vaccination for HPV for both sexes between ages 9-26 years
- Screening on Road safety and counselling on seat-belt use, drinking and driving
- Screening mammography examination every 1-2 years for women over 40 years
- Screening for domestic violence against women
- Colorectal cancer screening for patients over 50 annually, using faecal occult blood testing

2. Please indicate if you agree with the following expert recommendation from round 1 on periodic health check-ups.

During the first round, some experts who were in favour of conducting periodic health check-ups recommended that at least annual check-ups were feasible. Public health education on the importance of having periodic health check-ups can be provided easily using mass media; both private and public set-ups can be utilized; guidelines and protocols can be prepared on areas of check-ups; periodic health check-ups can be linked with the insurance system; health facilities need to allocate funds for health promotive purposes, and patients who have risk factors for certain diseases, such as cardiovascular disease, could highly benefit from the periodic health check-up approach. The experts who argued against having periodic health check-ups stated there would be a high possibility of cost problems related to the services, given the high out-of-pocket health expenditure existing in the country. In addition, these experts questioned what should be done if patients had positive results upon screening and could not afford the treatment or the health insurance system would not support the specific health problem? They added that health facilities and health professionals should be adequately ready in terms of numbers, skills, equipment and supplies to provide such promotive and preventive activities.

- Strongly Agree
- Agree
- Neutral
- Disagree
- Strongly Disagree

2.1 If you have any comment on part of the above description, please add.

3. Please mark only the areas you still think important to have case finding testing or screening (you may not necessarily agree with any of the options).

Case finding is testing or screening of patients for a condition other than the one for which they sought medical care. Some expert suggested case finding for breast cancer and risk for STIs needed to be included. These two clinical areas and previous case finding testing or screening lists on which more than 50% experts agreed but did not reach the consensus cut-off point (75%) are put forward below:

- Actively searching for obesity
- Finding smokers to provide counselling and care
- Actively looking for physical inactivity
- Actively looking for patients at risk of breast cancer
- Actively finding patients at high risk for STIs

4. Do you agree with the following recommendations, suggestions and justification forwarded by some experts on health promotion, disease prevention and treatment related to hypertension?

The experts emphasised that the perception of NCDs and their risk factors as a disease of the affluent is still predominant among health workers. The experts advised that health professionals need to strictly link the health promotion and disease prevention to curative service (such as advice on healthy diet, physical exercise and so on) routinely. For this role, health service providers’ skills and attitudes need to be built up and improved, and making high quality and functional equipment such as BP apparatus available is essential. In addition, health facilities need to prepare reference hand outs for the health workers that remind them not to avoid critical physical examination and measurements. Such reminders need to be posted in every outpatient and inpatient department as a standard activity. Enhancing patients’ awareness of the importance of available health promotion and disease prevention services is necessary to make each patient claim for critical preventive services whenever they are overlooked by health service providers. Some experts expressed concern that in health facilities where the patient load was high, there was a tendency among the health professional not to measure the blood pressure of patients who apparently looked well. The experts pointed out that routinely taking blood pressure was an easy and feasible activity. Despite the shortage of staff in some health facilities, taking vital signs should to be a must-do activity, something that should not be compromised. To ensure all patients’ blood pressure is measured, clinical audits must be regularly conducted in all health facilities. Therefore, in order to bring about change, the experts suggested that the regular follow-up by the health facility managers, which includes conducting patient chart audits, should increase measurement of blood pressure to patients. Regular inspection and maintenance of available BP measurement apparatus and routine sensitization sessions for health service providers on the importance of measuring blood pressure would improve the early detection of hypertension and prevention of its complications. The health facilities’ policy needed to address such preventive aspects and working guidelines should be prepared to make them practical. The experts pointed out the need to reorient the current health promotion and disease prevention approach in such a way that the monitoring system is strengthened and the health professionals’ responsibility and accountability at all level is ensured.

- Strongly Agree
- Agree
- Neutral
- Disagree
- Strongly Disagree

4.1 If you have any comment on part of the above description, please add below

--------------------------------------------------------------------------------------------------------------------

5. Do you agree with the following recommendations, suggestions and justification put forward by some experts on health promotion, disease prevention and treatment related to cardiovascular diseases?

The experts recommended increasing the Ethiopian population’s awareness of cardiovascular diseases and their risk factors which would help them to utilize preventive services. Making preventive risk assessment services available, especially for patients with high risk factors such as a family history, obesity, and hypertension, is very important. The experts advised further that preventive medicine needed to be well incorporated in health professionals’ training by revising curriculum of medical students. Providing in-service training, job aid for health workers to pick patients and their attendees at high risk of developing major cardiovascular diseases and stroke is also important. The addition of cardiovascular disease screening and key trigger questions in the existing treatment algorithms is advisable. Moreover, the inclusion of preventive services in health insurance packages also encourages screening for cardiovascular diseases and their risk factors for stroke. Activities that promote community’s healthy life styles such as sport activities, gyms and recreational places need to be expanded and availed within fair access to the community. Providing health education on dietary habits of individuals and availing essential technologies and medicines, such as EKG, Echocardiography, with reasonable price makes the preventable for cardiovascular diseases prevention service accessible.

- Strongly Agree
- Agree
- Neutral
- Disagree
- Strongly Disagree

5.1 If you have any comments on part of the above description, please add below

--------------------------------------------------------------------------------------------------------------------

6. Do you agree that measuring the blood cholesterol level of all patients (who had not been tested or were eligible for repeated cholesterol testing) should be included in patient care guidelines in the Ethiopian context?

Considering the guidelines that emphasize the importance of having a fasting cholesterol test for everyone every five years beginning at age 20 (Harvard Medical School 2009:3), the experts were asked if they agreed on measuring the blood cholesterol level of all patients (who had never tested or were eligible for repeated cholesterol testing) should be included in patient care guidelines in the Ethiopian context. Of the experts, 70% (n=14) agreed; while 20% (n=4) remained neutral, and 10% (n=2) disagreed.

- Strongly Agree
- Agree
- Neutral
- Disagree
- Strongly Disagree

7. Do you agree with the following recommendations, suggestions and justification forwarded by some experts on health promotion, disease prevention and treatment related to hyperlipidaemia diseases?

Asked about what recommendation they would suggest to improve the health facilities’ low performance in health promotion, prevention and effective treatment of hyperlipidaemia (elevated blood cholesterol level), they suggested there is a need for policy-level commitment (through evidence-based policy briefs and advocacy); in-service training for health professionals’ skills development and awareness; preparing guidelines and other supportive documents and materials in the health facilities for hyperlipidaemia prevention and treatment; improving the health facilities’ laboratory capacity to conduct the investigations, and integrating health professionals with other stakeholders, including nutrition experts, the food and nutrition society, and the Pharmaceutical Fund and Supply Agency (PFSA), to make the equipment and the test kits for performing fasting lipid profile available. The experts also suggested the government should stipulate and support medical equipment and supply importers to import the technologies and medical supplies for measuring blood cholesterol levels. Patients and individuals visiting the health facilities need to be made aware of the importance of measuring blood cholesterol levels on a regular basis to increase the demand for such services and obtain benefit from the service linking with the health care insurance system.

- Strongly Agree
- Agree
- Neutral
- Disagree
- Strongly Disagree

7.1 If you have any comment on part of the above description, please add below

--------------------------------------------------------------------------------------------------------------------

8. Do you agree with the following recommendations, suggestions and justification forwarded by some experts on health promotion, disease prevention and treatment related to cancer?

Health professionals’ knowledge on cancer screening, diagnosis and treatment was repeatedly mentioned as an area that required improvement. Some health experts pointed out that it is good to make the cancer screening affordable for the majority of the population and concomitantly make cancer treatment centres available. Cancer prevention approaches, such as taking the sexual history of women and screening for cervical cancer, need to be integrated in routine medical practices. Cancer investigations, such as mammography, colonoscopy, and PAP smear, need to be available at an affordable price to the general population and effectively utilized by health service providers.

- Strongly Agree
- Agree
- Neutral
- Disagree
- Strongly Disagree

8.1 If you have any comment on part of the above description, please add below

--------------------------------------------------------------------------------------------------------------------

9. Do you agree with the following recommendations, suggestions and justification forwarded by some experts on health promotion, disease prevention and treatment related to diabetes?

Asked for their recommendations, comments or experience for diabetes screening, the experts suggested that conducting blood sugar tests as a routine activity at clinics was easy and feasible, yet not widely practised, especially for children. The costs of treating diabetes and its complications were far higher than early detection and non-medical management of it. Some experts suggested that screening priority should be given to patients who have a high risk for diabetes, such as people with BMI > 25; a family history of diabetes or some genetic predisposition; people who lived unhealthy lifestyles and patients with other chronic NCDs.

- Strongly Agree
- Agree
- Neutral
- Disagree
- Strongly Disagree

9.1 If you have any comment on part of the above description, please add below

--------------------------------------------------------------------------------------------------------------------

10. Do you agree with the following recommendations, suggestions and justification forwarded by some experts on health promotion, disease prevention and treatment related to cigarette smoking?

The experts pointed out that awareness of the magnitude of cigarette smoking as a public health problem in our country was low, but its prevalence needed to be established in research. The integration of cigarette smoking rehabilitative services in health systems is essential, even if only done in selected health facilities. Health education on the health hazards of smoking, professional counselling services for addicted patients, and facilitating peer-to-peer discussion to quit smoking were more feasible in Ethiopian health facilities set-ups. START HERE Therefor e, in Ethiopia context, integrating cigarette smoking rehabilitation unit, for disease related to cigarette smoking addiction, is feasible only if performed at selected health facilities.

- Strongly Agree
- Agree
- Neutral
- Disagree
- Strongly Disagree

10.1 If you have any comment on part of the above description, please add below

--------------------------------------------------------------------------------------------------------------------

11. Do you agree that in the Ethiopian context, laboratory tests to test and monitor blood alcohol level should be initiated in all health facilities but only selected health facilities should provide an established rehabilitation unit that helps to quit from alcoholism?

During the first round discussion, the experts were asked if in the Ethiopian context, rehabilitation units at health facilities for diagnosis and treatment of harmful use of alcohol were advisable. The experts did not consensus because 70% (n=14) agreed; 20% (n=4) remained neutral, and 10% (n=2) disagreed. The question was then modified as above in the second round.

- Strongly Agree
- Agree
- Neutral
- Disagree
- Strongly Disagree

12. Do you agree with the following recommendations, suggestions and justification forwarded by the experts on health promotion, disease prevention and treatment related to harmful use of alcohol?

The experts emphasised that alcohol producing companies were flourishing in the country with virtually unrestricted advertisement in all the media to encourage alcohol consumption. Legislation should be introduced and implemented to limit advertising and selling, as well as prohibit selling to underage children. Alcohol drinking is one of the major causes of road traffic accidents therefore alcohol detection for drunk driving needs to be strictly applied. Moreover, there is a need to establish a unit at health facilities to treat alcohol withdrawal. Health education on alcohol, including addiction and its psycho-social problems, drug interaction, as a cause of liver diseases is not commonly provided. Mass media, health professionals and spiritual leaders can play major role in this aspect.

- Strongly Agree
- Agree
- Neutral
- Disagree
- Strongly Disagree

12.1 If you have any comment on part of the above description, please add below

--------------------------------------------------------------------------------------------------------------------

13. Do you agree with the following recommendations, suggestions and justification by the experts on health promotion, disease prevention and treatment related to physical exercise?

In phase 1, some experts advised that it was expensive and not feasible to have physical exercise and rehabilitation units in all health facilities. Only hospitals needed to have equipment and exercise devices for rehabilitation purposes. For the general population and sick patients, however, there was a serious need of expansion for affordable physical exercise sites, gyms and physiotherapy units in Addis Ababa.

- Strongly Agree
- Agree
- Neutral
- Disagree
- Strongly Disagree

13.1If you have any comment on part of the above suggestions, please add below

--------------------------------------------------------------------------------------------------------------------

14. Thank you for completing the questionnaire. Please provide any feedback or final comments on the study you may have below.

--------------------------------------------------------------------------------------------------------------------
